# Electrochemical Deposition Treatment (EDT) as a Comprehensive Rehabilitation Method for Corrosion-Induced Deterioration in Concrete with Various Severity Levels

**DOI:** 10.3390/s21186287

**Published:** 2021-09-19

**Authors:** Je-Kyoung Kim, Jurng-Jae Yee, Seong-Hoon Kee

**Affiliations:** 1 National Core Research Center for Disaster-Free and Safe Ocean Cities Construction, Dong-A University, Busan 49315, Korea; kjktit@dau.ac.kr; 2 Department of ICT Integrated Ocean Smart Cities Engineering, Dong-A University, Busan 49315, Korea

**Keywords:** corrosion, wetting-and-drying cycles, MgCl_2_, electrochemical deposition, electrochemical impedance spectroscopy

## Abstract

The primary purposes of this study are to investigate the feasibility of electrochemical deposition treatment (EDT) as a comprehensive rehabilitation method for corrosion-induced deterioration in reinforced concrete with various severity levels, and to propose a guideline for the determination of critical factors to advance EDT. This study includes three experimental phases, each of which simulates the initiation (de-passivation), propagation (high corrosion activity), and acceleration (formation of a surface-breaking crack) periods of corrosion-induced deterioration. After completion of a series of accelerated corrosion tests, damaged concrete samples with different severity levels are rehabilitated by a series of EDT processes using a MgCl_2_ solution in an electrolyte. The main variables for this experiment are the concentration levels (0, 0.3, 1.0 and 3.0 M) of a MgCl_2_ solution for test phase 1, charging time (0, 2, and 7 days) in EDT for test phase 2, and configuration of pre- and post-treatment processes in EDT for test phase 3. The rehabilitation performance of EDT is evaluated by analyzing the AC impedance properties of the steel-and-concrete interface using electrochemical impedance spectroscopy (EIS) for the test phases 1 and 2, and microscopic alternation in concrete cracks using optical microscopic image and SEM/EDX. It is demonstrated that EDT is an effective method for preventing and mitigating corrosion-induced deterioration in the initiation and rust propagation periods of corrosion and for repairing (closing and filling) a corrosion-induced surface-breaking crack in the acceleration phase of corrosion. Corrosion-resistant performance of concrete increases as the concentration levels of a MgCl_2_ solution in an electrolyte increases and as the charging time in EDT increases. In addition, a post-treatment process (applying a NaOH solution) after the electrochemical deposition process significantly improves crack-repairing performance of EDT.

## 1. Introduction

**Figure 1:**
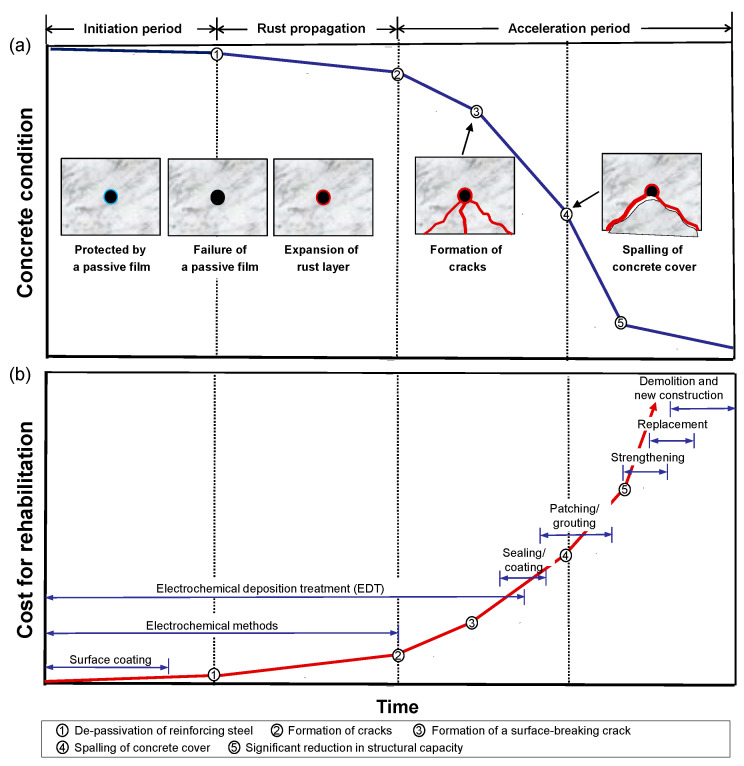
Conceptual illustration of typical deterioration types in reinforced concrete caused by steel corrosion and various rehabilitation methods: (**a**) deterioration process of steel corrosion in concrete and (**b**) effective rehabilitation methods for deteriorated concrete with various severity levels.

Corrosion is a process through which metals in manufactured states return to their natural oxidation states. Corrosion of steel in reinforced concrete is generally described by multiple phases, including initiation, rust propagation, and acceleration as illustrated in Figure 1a [1,2,3]. Due to the high alkalinity of concrete (pH 12~13), the reinforcing steel in sound concrete forms a thin passive layer (iron oxide film, Fe_2_O_3_) on its surface that significantly reduces the corrosion rate. The passive film becomes unstable when the pH of the concrete lowers below a certain level or the chloride ion concentration in concrete exceeds a threshold level [4]. The failure of the passive film would lead to corrosion of reinforcing steel in concrete exposed to the corrosive environment with sufficient moisture and oxygen. Growth of the surface rust layer may cause microcracks in concrete due to excessive internal tensile stresses caused by the expanded volume of the rust layer [5]. The enhanced microcracks and internal voids increase the permeability of concrete, thereby accelerating the penetration rate of moisture and harmful substances in the concrete. Without proper management actions, it can result in serious concrete damages (e.g., surface cracking, delamination and/or spalling of concrete), which lead to degradation of strength and/or rigidity of structural members.

Corrosion of infrastructures causes high costs to the society of many industrialized countries in the world. Estimated annual direct corrosion cost is about US $240 billion in the US. Approximately 20% of the direct costs were attributed to corrosion of reinforced concrete (highway bridges, parking garages, retaining walls, tunnels, etc.) [6]. More than 55% of structures’ repairs were caused by steel corrosion in Europe [1]. The corrosion cost of the damaged infrastructure is up to 3.8% of the gross domestic product (GDP) of developed countries, and up to 5.2% in the Middle East [7,8,9]. In Korea, corrosion cost was calculated as 2.9% of GDP [10]. From the perspective of infrastructure management agencies, it is of importance to find effective ways for maintenance of old and/or damaged reinforced concrete structures with corrosion issues, which keeps the structures in sound condition and prolongs their service life, thereby reducing social cost for maintenance of infrastructure systems.

There are various practical methods for rehabilitation of old and/or damaged reinforced concrete structures that suffer from different phases of corrosion-induced deterioration (see Figure 1b). Before corrosion activity initiates (phase 1), there are several corrosion protection and prevention methods that can improve the corrosion-resistant properties of concrete. One example is corrosion inhibitors, which are mixed with fresh concrete for new construction and penetrate hardened concrete in existing structures. The risk of steel corrosion can be controlled by using alternative reinforcing steel (corrosion resistant steel, fiber-reinforced plastic, coated steel, etc.) and concrete coating. However, it is known that the corrosion protection effects of these conventional methods become ineffective once corrosion of steel in concrete initiates. In the rust propagation phase (phase 2), it has been demonstrated that electrochemical methods (e.g., cathodic protection, electrochemical chloride removal and electrochemical re-alkalization) are most effective for suppressing corrosion activity and mitigating corrosion-induced deterioration in reinforced concrete. Cathodic protection method is regarded as the only rehabilitation technique that suppresses the corrosion activity of reinforced concrete regardless of concentration levels of chloride ions in concrete [11]. When a fracture process of concrete initiates in the acceleration phase of corrosion (phase 3), mechanical repairing of concrete is necessary, along with corrosion mitigation techniques based on the electrochemical methods. There are various repairing methods for improving durability performance of concrete: epoxy injection, routing and sealing, grouting, patching, and jacketing and casing. Those conventional methods have been demonstrated to be successful for improving permeability of concrete and mechanical performance of distressed concrete. However, conventional repair methods are not always useful for repairing early stages of corrosion-induced microcracks in concrete, and/or they can be labor- and cost-intensive. In summary, conventional rehabilitation methods in practice have their advantages in specific phases of corrosion levels, but still have some limitations to be used as a comprehensive rehabilitation method for corrosion-induced deterioration with a wide range of severity levels (see Figure 1b).

Electrochemical deposition treatment (EDT) is a kind of cathodic protection method. After being proposed in Japan first in early 1990s [12,13], there has been numerous researchers demonstrating the effectiveness of EDT for rehabilitation of corrosion-induced deterioration in concrete with various severity levels. EDT forms precipitates in microstructures of concrete (voids in cement pastes, steel-concrete interface and aggregate-cement paste interface), which results in corrosion-resistant concrete [14,15,16,17,18,19]. Like the conventional cathode protection method, EDT is effective for reducing chloride ion concentration in concrete exposed to high chloride environment [17,18,20]. For the propagation period, it was demonstrated that EDT re-passivates corroded rebars by re-alkalizing concrete in the vicinity of corroded steel, which suppresses corrosion rate. In addition, many researchers demonstrated the unique advantages of EDT as a rehabilitation of distributed surface-breaking cracks in concrete, which cannot be effectively recovered by conventional crack repair techniques [14,15,17,18,19,21,22,23,24,25,26]. EDT improves mechanical properties of corroded reinforced concrete: bonding-strength steel and concrete, flexural capacity of reinforced concrete beams. In addition, there are various theoretical and analytical models that explain crack repairing process using EDT [27,28,29,30]. Therefore, EDT is a high-potential rehabilitation method that is applicable to deteriorated concrete with a wide range of severity levels. However, there are still several research topics that have not been completely addressed in the literature on determination of some critical factors (i.e., concentration level of cations in an electrolyte, charging time and configuration of EDT) towards an optimum EDT process. 

In this study, a series of experimental studies was performed in the laboratory to investigate the effectiveness of EDT for rehabilitation of deteriorated reinforced concrete with three different corrosion levels: initiation, rust-propagation, and acceleration phases of steel corrosion. Each test phase was composed of multiple experimental processes that include preparation of test specimens, accelerated corrosion tests, electrochemical deposition treatment (EDT), and condition assessment of solid, deteriorated, and rehabilitated reinforced concrete specimens. The main variables of this study include the concentration levels (0, 0.3, 1.0 and 3.0M) of a MgCl_2_ solution for test phase 1, charging time of EDT (0, 2, and 7 days) for test phase 2, and configuration of pre- and post-treatment processes in EDT for test phase 3. As will be discussed, the experimental results in this study would provide a fundamental basis to better understand and further improve the capability of the EDT method as a comprehensive rehabilitation method of corrosion-induced deterioration with various severity levels. 

## 2. Experimental Methods

### 2.1. Preparation of Solid Concrete Specimens

**Figure 2:**
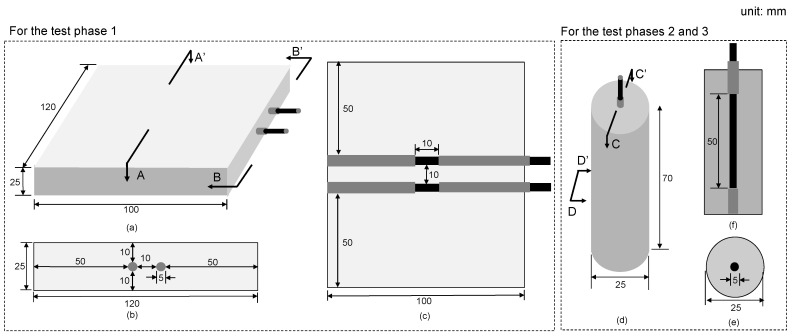
Configuration and dimensions of concrete samples used for the test phases 1, 2 and 3: (**a**–**c**) for isometric view, sectional view A-A’ and sectional view B-B’ for the concrete prism samples used in test phase 1, and (**d**–**f**) for isometric view, sectional view C-C’ and D-D’ for the concrete cylinder samples used in test phases 2 and 3.

Test phase 1 used four concrete prism samples (100 mm length × 120 mm width × 25 mm thickness) shown in Figure 2a–c. Two reinforcing steel bars (rebars), 5 mm in diameter and 100 mm long, were embedded at the center of the sample. The reinforcing bars were insulated with a heat shrink tube with a diameter of 45 mm and B-coated agent on both ends. The middle 10 mm length was directly exposed to the concrete. Therefore, the effective area of a rebar was fixed at 3.14 cm^2^. The cuboidal concrete samples were made of Type I Portland cement and ISO standard sand with a water-to-cement ratio of 0.6 and a cement-to-sand ratio of 0.5. A total of four samples were fabricated under the same shape and rebars. The rebar spacing was kept constant at 10 mm in all samples. The clear cover of rebars was kept constant at 10 mm. 

In contrast, the test phases 2 and 3 used concrete cylinder samples (25 mm diameter by 70 mm length) shown in Figure 2d–f. A total of eight identical concrete cylinders were fabricated by the same way used for the concrete prisms. Four of the eight concrete cylinders were used for the test phases 2 and 3, respectively. A single reinforcing bar, 5 mm diameter and 100 mm length, was embedded at the center of the concrete cylinders. The middle 50 mm of a rebar was directly exposed to concrete, resulting in the effective area of 7.85 cm^2^. 

### 2.2. Preparation of Deteriorated Concrete Specimens

**Figure 3:**
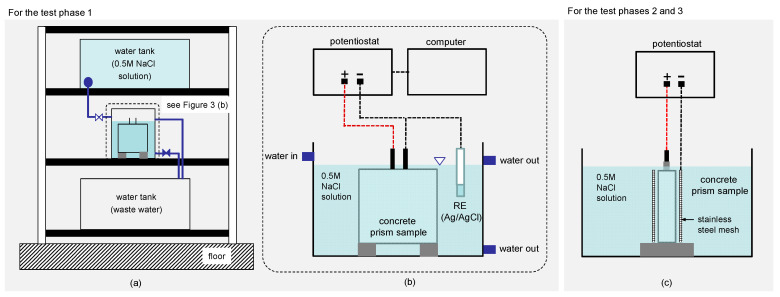
Illustration of test setups for the accelerated corrosion test: (**a**,**b**) the test setup for the wetting-and-drying cycles for the test phase 1, and (**c**) test setup for the impressed current technique for the test phases 2 and 3.

For test phase 1, the four concrete prism samples (TP1-1, TP1-2, TP1-3 and TP1-4 in Table 1) were exposed to wetting-and-drying cyclic environment to accelerate corrosion of rebars in concrete. A wetting-and-drying refers to the process of immersing the concrete specimens in 0.5M NaCl solution for 8 h and dried at atmosphere under room temperature for 16 h. The NaCl solution was prepared using analytical grade NaCl (99% purity, Daejung, Korea). The timing of wetting-and-drying was automatically controlled by two programmable valves (inlet and outlet, respectively) in a test setup developed at Dong-A University (see Figure 3a,b). For the wetting process, the outlet valve was close, and the inlet valve was opened to fill a water tank to the designed water level. An additional outlet was installed at the designed water level to drain excessive water. For the drying process, the outlet valve was opened to drain water, and the concrete sample surfaces were exposed to atmosphere and air-dried. In this study, the wetting-and-drying cycles were repeated for a total 45 cycles. 

For test phase 2, corrosion of rebars in the three of the four concrete cylinders (TP2-2, TP2-3, TP2-4 in Table 1) was accelerated by the impressed current technique [31] to induce high corrosion activity and simulate the expansion of rust layers in concrete. One concrete cylinder, TP2-1, was used as control specimen, representing sound concrete in test phase 2. Figure 3c shows the test setup for the impressed current technique. A concrete cylinder was placed in a water tank so that the top surface of the concrete was completely submerged in the 0.5M NaCl solution prepared using analytical grade NaCl (99% purity, Daejung, Korea). The concrete surface was wrapped around by titanium mesh with a height of 70 mm, which serves as a cathode in the accelerated corrosion setup. Direct current with a density of 0.5 μA/cm^2^ was impressed to three cylindrical concrete specimens for 24 h by a potentiostat (Won-A tec. ZIVE MP2, Seoul, Korea). For test setup 3, the accelerated corrosion test setup and procedure were the same as those from test setup 2, except for applying an enhanced current density of 1 mA/cm^2^ for 48 h to generate corrosion-induced surface-breaking cracks in concrete. Table 1 summarizes the accelerated corrosion test methods for test phases 1, 2 and 3. 

### 2.3. Rehabiliation of the Deteriorated Concrete Samples by EDT

**Figure 4:**
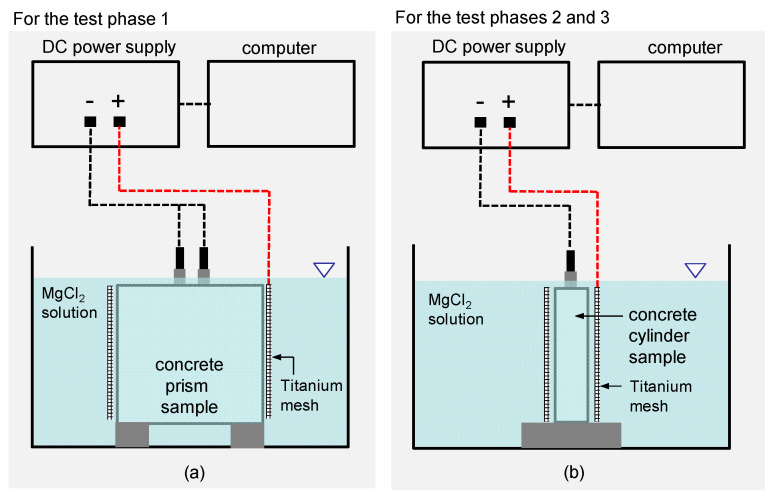
Illustration of the test setups of electrochemical deposition treatment (EDT) for: (**a**) concrete prism samples in test phase 1 and (**b**) concrete cylinder samples in test phases 2 and 3.

Figure 4a,b illustrate the test setups of the electrochemical deposition treatment (EDT) for rehabilitation of deteriorated concrete prisms for test phase 1 and concrete cylinders for test phases 2 and 3, respectively. The two test setups are composed of a potentiostat, an anodic electrode (titanium mesh), a cathodic electrode (rebars in concrete), and an electrolyte (a MgCl_2_ solution). A potentiostat was used to pass a constant electric current, −2 μAcm^−2^, between the embedded rebar (cathodic electrode) in concrete and a titanium anode (anodic electrode) in a MgCl_2_ solution. The stabilized potential levels measured in the EDT processes was in a range of −0.8 V to −1.0 V (vs. Ag/AgCl), which prevented the formation of hydrogen gas around rebars in concrete (cathode) during EDT [32,33]. Table 2 summarizes configuration of EDT processes used in the test phases 1, 2 and 3. The main variables in this study include the concentration levels (0, 0.3, 1.0 and 3.0 M) of a MgCl_2_ solution for test phase 1, the duration of polarization (0, 2 and 7 days) during EDT for test phase 2, and the configuration of pre- and post-treatment processes in EDT for test phase 3.

In test phase 1, EDT was applied to the four concrete prisms after completion of the 13th cycle of the wetting-and-drying process (see Figure 4a). The timing of the EDT was determined based on observations in the previous research [34]. It required at least 13 cycles of the wetting-and-drying process for the corrosion rate (=1/*R_P_*, *R_P_* is polarization resistance of reinforced concrete samples) to exceed a criterion for the initiation of corrosion (i.e., failure of a passive film on the surface of a reinforcing steel) (4 × 10^−6^ Ω^−1^cm^−2^) [35]. Four different concentrations of MgCl_2_ solutions (0M, 0.3M, 1M, and 3M) were used to investigate the effect of concentration level of MgCl_2_ in an electrolyte on the corrosion prevention capability of the EDT. After completion of EDT, all concrete samples were taken out of the water tank and oven-dried under 60 °C. The dried samples were exposed to the wetting-and-drying cycles (from 14th to 45th cycles) again to investigate the corrosion-resistant properties of the concrete prisms after EDT. 

In test phase 2, electrochemical deposition was applied to deteriorated concrete cylinders with high corrosion activity (see Figure 4b). Three deteriorated concrete specimens were immersed in a separate water tank with a 2M MgCl_2_ solution. A constant current with a density of −2 µAcm^−2^ was continuously applied for 0, 2, and 7 days to investigate the effect of polarization time on the rehabilitation performance of EDT. 

In addition, EDT was used to repair a surface-breaking crack in the four concrete cylinders in test phase 3. In this study, several extra processes were added before and after the main EDT process to improve the crack-filling capability of EDT. The multi-stage EDT is composed of four processes, including pre-treatment, electrical deposition, post-treatment, and baking. In the pre-treatment stage, the cracked concrete cylinders were submerged in NaOH solution (pH 13) for 10 h. In the main EDT stage, concrete samples were immersed in a 2M MgCl_2_ solution with a constant current density of −2 µAcm^−2^ for 240 h (10 days). After that, treated concrete cylinders were submerged again in a 2M NaOH solution (pH 13) for 10 h as a post-treatment. Test phase 3 included four different scenarios of EDT processes to investigate the effect of pre- and post-treatment on the crack-filling capability of the multi-stage EDT.

### 2.4. Electrochemcial Impedance Spectroscopy (EIS)

**Figure 5:**
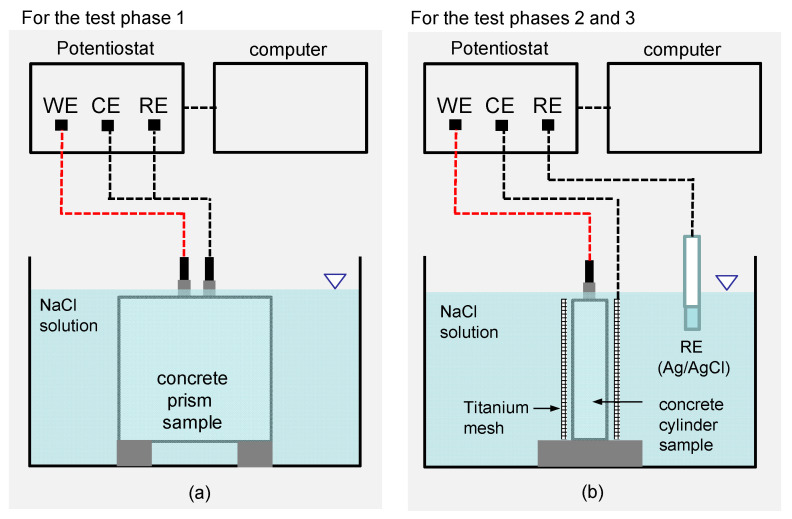
Illustration of test setups for electrochemical impedance spectroscopy (EIS) using: (**a**) the two-electrode system for the concrete prism samples in the test phase 1, and (**b**) the three-electrode system for the concrete cylinder samples in the phase 2.

Electrochemical impedance spectroscopy (EIS) was used to investigate the effectiveness of EDT processes on the rehabilitation of deteriorated concrete samples in the test phases 1 and 2. Figure 5a,b illustrate the test setups of EIS using two- and three-electrodes, respectively, each of which was applied to the concrete prism samples and cylinder samples. In the two-electrode system, one rebar in a concrete prism sample was connected to the working electrode (WE) terminal in a potentiostat, while the other rebar was connected to the counter electrode (CE) and the reference electrode (RE) terminal (Figure 5a). In contrast, the three-electrode system used a rebar as a WE, a titanium mesh as a CE, and the silver chloride electrode (Ag/AgCl) as an RE. It was demonstrated that the two-electrode system is effective to measure the electrochemical impedance properties of concrete in the wet-and-dry cycling conditions [34,36]. For both the two- and three-electrode systems, AC impedance of concrete was measured by a potentiostat (Won-A tec. ZIVE MP2, Seoul, Korea), which passed alternating voltages of ±10 mV under a frequency range from 1 mH to 100 kHz. 

**Figure 6:**
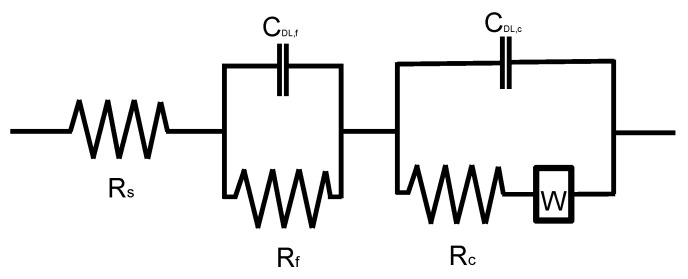
Equivalent electrical circuit models for modelling corrosion of steel in concrete considering electrochemical deposit in concrete.

In this study, a reinforced concrete sample was modelled by an equivalent EIS circuit shown in Figure 6, which is composed of three impedance layers [7]. Resistance components of each layer (i.e., *R_s_*, *R_f_*, and *R_c_*) are connected in series, where *R_s_* represents the electrical resistance associated with the electrolyte solution in concrete pores; *R_f_* refers to the resistance of interfacial film on a steel rebar caused by rust propagation and/or precipitation of cement hydration and electrochemical deposition [37]; *R_c_* corresponds to the resistance of the charge transfer involved in the corrosion mechanism. Impedance, (*Z_CPE_*), caused by capacitance actions in the interface of the layers, was modelled by the constant phase element (CPE), which was connected in parallel to a resistance component in each layer.

(1) $$Z_{CPE,i}=\frac{1}{C_{CPE,i}{\left(jw\right)}^{\alpha_{i}}}$$

where *C_CPE,i_* is the electric capacitance, *α_i_* is a constant for calibrating the non-uniform properties of steel and concrete interfaces, the subscript *i* represents an interface where the capacitance action occurs (*f* and *d* stands for the interfacial film layer and the double layer, respectively), *j* is complex number and *w* is the angular frequency (=2π*f*, *f* is frequency). Warburg impedance element (*W*) was used to model the impedance due to diffusion of corrosion product (*Z_W_*), which is composed of a resistance component *R_W_*, a capacitance component τ and a calibration factor *α_W_*.

(2) $$Z_{W}=\frac{R_{W}\tan h\left[{\left(jw\tau \right)}^{\alpha_{W}}\right]}{{\left(jw\tau \right)}^{\alpha_{W}}}$$

Finally, the total impedance (*Z*) of the EIS circuit in this study is represented as follows,

(3) $$Z=R_{s}+\frac{R_{f}}{1+R_{f}/Z_{CPE,f}}+\frac{R_{c}+Z_{W}}{1+(R_{c}+Z_{W})/Z_{CPE,d}}$$

The critical parameters were determined by fitting experimental data to equivalent model by using commercially available program ZView^®^. Corrosion rate is defined as a reciprocal of the polarization resistance, *R_P_* [38]. In this study, corrosion rate was defined as a reciprocal of *R_c_*, which is equivalent to *R_P_*, as follows,

(4) $$corrosion\;rate=\frac{1}{R_{c}}$$

By inspection of Equation (3), *Z* converges to *R_s_* as *w* increases to infinite, while *Z* goes to *R_s_* + *R_c_* + *R_f_* + *Z_W_* as *w* decreases to zero as follows,

(5) $$Z_{high}=R_{s}$$

(6) $$Z_{low}=R_{s}+R_{c}+R_{f}+Z_{W}$$

In this study, the polarization resistance, $R_{P}$, was simply expressed as the difference between high frequency impedance, *Z_high_*, and low frequency impedance, *Z_low_*, provided that *R_c_* value is large enough compared to *R_f_* and *Z_W_*. Consequently, the approximate polarization resistance $R_{P}^{\prime }$ can be expressed as follows,

(7) $$R_{P}^{\prime }=Z_{low}-Z_{high}\;≅R_{c}$$

It was demonstrated that this simplified approach is effective for rapid monitoring of the corrosion rate by EIS [34,39].

## 3. Results and Discussion

### 3.1. Test Phase 1: Recovery of Corrosion Protection Performance in the Corrosion Initiation Phase

**Figure 7:**
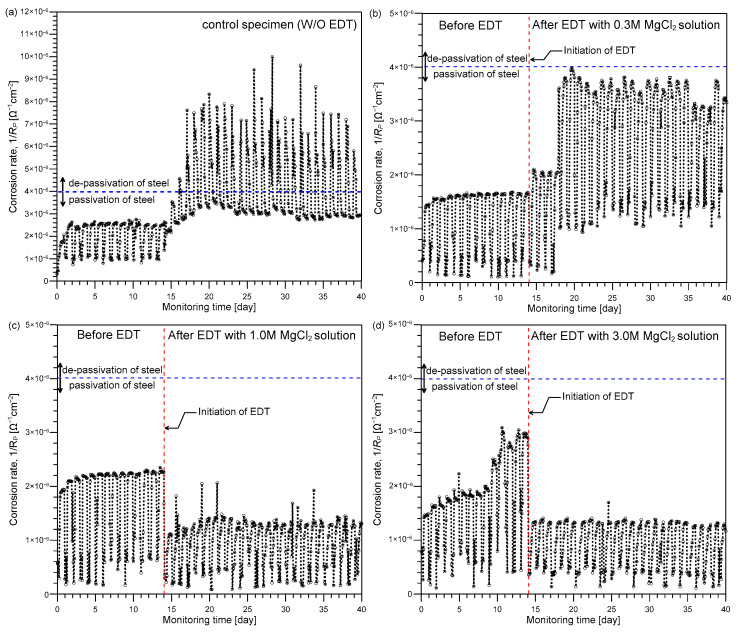
The variation of corrosion rates in the four concrete prism samples in test phase 1: (**a**) the control sample without EDT, TP1-1, (**b**–**d**) the three rehabilitated samples treated by EDT with three different concentration levels of MgCl_2_ solution with: (**b**) 0.3M, (**c**) 1M and (**d**) 3M, respectively.

**Figure 8:**
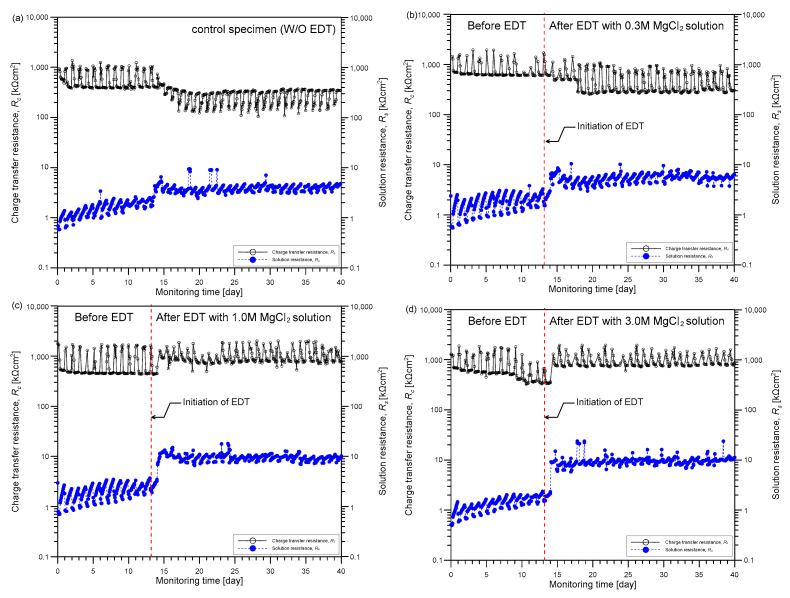
The variation of charge transfer resistance *R_c_* and the solution resistance *R_s_* in the four concrete prism samples in test phase 1: (**a**) the control sample without EDT, TP1-1, (**b**–**d**) the three rehabilitated samples treated by EDT with three different concentration levels of MgCl_2_ solution: (**b**) 0.3M, (**c**) 1M and (**d**) 3M, respectively.

Figure 7 shows the variation of corrosion rate in the four concrete prism samples exposed to a total 45 cycles of wetting-and-drying cycles in test phase 1. The instantaneous corrosion rate in Figure 7 was determined based on the approximate polarization resistance $R_{P}^{\prime }$ in Equation (7). The impedance values at the low and high frequencies (i.e., $Z_{low}$ and $Z_{high}$) were determined at 10 mHz and 10 kHz, respectively [34]. Figure 8 shows the variations of $Z_{low}$ and $Z_{high}$ used to calculate $R_{P}^{\prime }$ in Figure 7. After completion of the 13th cycle of wetting-and-drying, three of the four concrete prism samples (TP1-2, TP1-3, and TP1-4) were rehabilitated by the EDT process (see Figure 4a). Test results obtained from the control specimen without EDT (TP1-1) are shown in Figure 7a, and test results from the rehabilitated concrete samples (TP1-2, TP1-3, and TP1-4) by EDT with three different MgCl_2_ concentrations (0.3M, 1M, and 3M) are shown in Figure 7b–d. The timing of EDT is indicated by red dash lines in Figure 7b–d. 

In the 1st~13th cycles of the immersion and drying cycles, the instantaneous corrosion rate in the four concrete prism samples was lower than the criterion for the de-passivation of reinforcing steel (4.0 × 10^−6^ Ω^−1^cm^−2^) [38]. After the 14th cycle, the corrosion rate of the control sample, TP1-1, suddenly increased over 3.0 × 10^−6^ Ω^−1^cm^−2^ in the dry condition and over 1 × 10^−5^ Ω^−1^cm^−2^ (about 250% of the de-passivation criteria) in the wet condition (Figure 7a). However, it was clearly observed in Figure 7b,c that the corrosion rate of concrete samples treated by electrochemical deposition (EDT) was controlled under the de-passivation level (i.e., 4.0 × 10^−6^ Ω^−1^cm^−2^) even after being continuously exposed to a harsh environment during the 14th to 45th cycles. The use of 0.5M MgCl^2^ solution was effective for prevention of steel corrosion by suppressing the corrosion rate under the de-passivation criterion (4.0 × 10^−6^ Ω^−1^cm^−2^). Specifically, Figure 8b shows that EDT was effective for improving *R_c_* and *R_s_* values in the steel and concrete interface. The recovery of corrosion protection performance by EDT is mainly attributed to the presence of precipitates on the steel surface. The precipitates formed by EDT impede charge transfer rate for the steel corrosion reaction (Fe → Fe^2+^ + 2e^−^) and reduction rate of dissolved oxygen (O_2_ + 2H_2_O + 4e^−^ → 4OH^−^) in the rust layer. Furthermore, the use of a higher concentration level of MgCl_2_ solution (1M and 3M) further decreased the corrosion rate of reinforced steel in concrete compared to the use of 0.3M MgCl_2_. Figure 7c,d shows that the corrosion rate of concrete samples treated by EDT in 1M and 3M MgCl_2_ solutions (TP1-3 and TP1-4) decreased to approximately 50% of the values before EDT process. However, the use of 3M MgCl_2_ solution does not show a clear benefit compared to 1M MgCl_2_ solution. The concrete samples in the experimental phase 1 were under low corrosion activity. Therefore, the EDT process can be governed by the amount of hydroxide ion (OH^−^) caused by corrosion of steel rather than the amount of magnesium ion (Mg^2+^) in concrete pore solution. In this study, it can be inferred that 1M MgCl_2_ is sufficient to recover corrosion protection capability of concrete under relatively low corrosion rate.

### 3.2. Test Phase 2: Recovery of Corrosion-Resistant Performance in the Rust Propagation Phase

**Figure 9:**
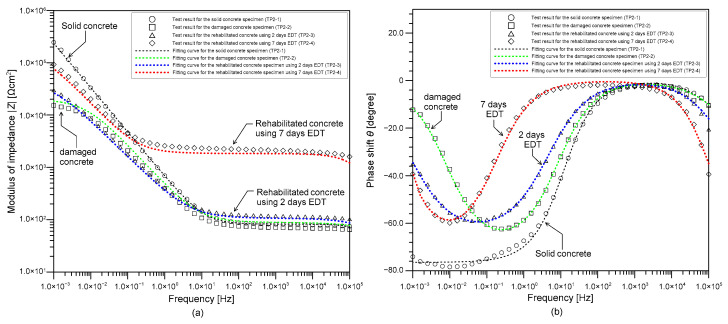
Experimental bode diagram and curve fitting for reinforcing steel in 0.5M NaCl solution: (**a**) modulus of impedance and (**b**) phase shift of impedance with (○) passive rebar in the concrete sample TP2-1, (□) corroded rebar in the concrete sample TP2-2, (◊) electro-deposited rebar with −2 µAcm^−2^ for 2 days in the concrete sample TP2-3, (Δ) electro-deposited rebar with −2 µAcm^−2^ for 7 days in the concrete sample TP2-4, respectively.

Figure 9a,b show the bode plots representing the variation of modulus and phase shift of AC impedance, respectively, with frequency measured on the four concrete cylinder samples in the test phase 2. The EIS spectra of the concrete specimens were measured under a frequency range of 1 mHz to 100 kHz with an amplitude of ±10 mV in a 0.5M NaCl solution using the three-electrode system (see Figure 5b). The bode plots measured on the solid and deteriorated concrete cylinder samples (TP2-1 and TP2-2) are presented as open circles (○) and open squares (□), respectively. Bode plots of the rehabilitated concrete samples by EDT for 2 days and 7 days (TP2-3 and TP2-4) are presented as diamonds (◊) and triangles (Δ), respectively. In addition, the optimum EIS curves predicted by the EIS equivalent circuit shown in Figure 6 are represented by dashed lines in Figure 9a,b. Results show that experimental data are in good agreement with the fitted curves. Table 3 summarizes the predicted EIS parameters from the curve fitting. 

Bode plots obtained from EIS are informative on the variation of interfacial conditions of steel and concrete in the concrete samples during EDT. The phase shift curves in Figure 9b shows that there are valleys in a low frequency region (100 Hz to 0.001 Hz). There are the other valleys appearing in the high frequency regions (100 kHz~10 kHz). The presence of valley in a phase shift curve of EIS spectra is physically interpreted as an impedance by storing electrons (i.e., capacitor action) in the interfaces between various component materials (e.g., steel, aggregates, and cement hydration products) in reinforced concrete [40]. The valley in the low frequency range is related to the double layer in the steel and concrete interface, while the valley in the high frequency range is related to an interfacial film representing rust propagation and precipitation of Ca(OH)_2_ [7,37]. It was noticed in Figure 9b that the slope of the curve becomes steeper as the polarization time increases in the EDT process. Therefore, it can be inferred that the valley in high frequency region also characterizes the precipitation of Mg^2+^ ions due to EDT. The modulus of impedance in a high frequency range of 10 kHz to 100 Hz (see Figure 9a), where the phase shift values are almost zero, represents the impedance caused by rust layer and precipitations on the steel surface (i.e., *R_s_*). In contrast, the modulus of impedance values in a low frequency range (0.01 Hz to 0.001 Hz) represents the impedance of charge transfer associated with corrosion reaction (i.e., *R_c_*). 

**Figure 10:**
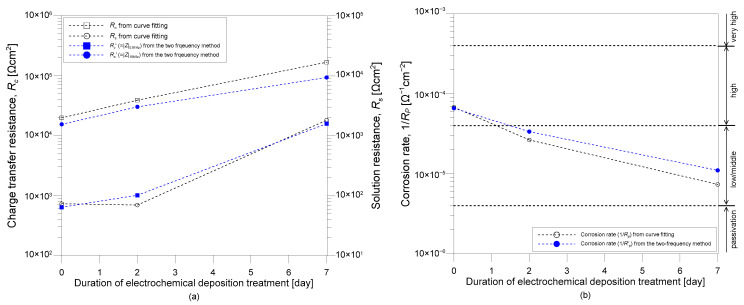
Variation of electrochemical impedance spectroscopy (EIS) parameters with duration of electrochemical deposition treatment (EDT): (**a**) variation of charge transfer resistance (on the left side in the y axis) and solution resistance (on the right side in the y axis) with duration of EDT, and (**b**) variation of corrosion rate with duration of EDT.

Figure 10 shows the variation of EIS parameters of the three concrete samples (TP2-2, TP2-3, and TP2-4) after being rehabilitated by EDT with different polarization times (i.e., 0, 2, and 7 days). Figure 10a shows the variation of charge transfer resistance *R_c_* (on the left side in the y axis) and solution resistance *R_s_* (on the left side in the y axis). The variation of the corrosion rate is shown in Figure 10b. Corrosion rate was determined by inserting *R_c_* values summarized in Table 3 into Equation (4). For comparison, the approximate polarization resistance $R_{P}^{\prime }$ based on the two frequencies was also shown in Figure 10. After completion of the accelerated corrosion test *R_c_* value of the concrete sample (TP2-2) was only 19.5 kΩcm^2^, which is significantly lower than the value of solid concrete sample (TP2-1). Accordingly, the corrosion rate of the TP2-2 sample was 5.12 × 10^−5^ Ω^−1^cm^−2^, indicating the deteriorated concrete before EDT is under high corrosion activity [38]. After EDT, *R_c_* value increases to 38.5 kΩcm^2^ and 165.1 kΩcm^2^ as the polarization time of EDT increases to 2 days and 7 days, respectively. Consequently, the corrosion rate of concrete sample decreases as the duration of EDT increases to 0, 2, and 7 days. The speed of corrosion rate becomes slower as the duration of EDT increases. Furthermore, it appears to converge into a certain value greater than a criterion for the initiation of corrosion (4 × 10^−6^ Ω^−1^cm^−2^). It can be inferred that there is a limitation in the status of deteriorated concrete that can be recovered by EDT processes, which is dependent on corrosion activity levels. 

### 3.3. Test Phase 3: Crack-Filling Capability of EDT 

**Figure 11:**
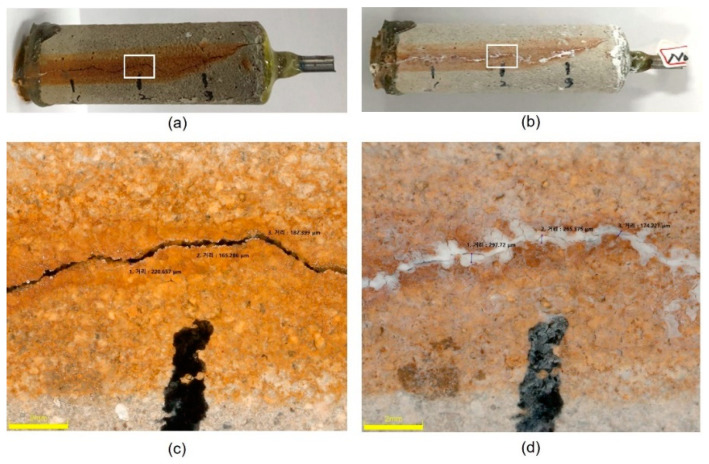
Optical microscope images of a surface-breaking crack formed in the concrete sample TP3-1: (**a**) before and (**b**) after being rehabilitated by EDT process, respectively, and (**c**,**d**) enlarged images in the region of interests pointed out by the white rectangular symbol in Figure 11a,b, respectively.

This section describes the efficiency of EDT on repairing (closing and filling) a surface-breaking crack in concrete samples. Figure 11a shows an optical microscope image of a surface-breaking crack formed in the concrete specimen (TP3-1) by the accelerated corrosion test. Figure 11c, an enlarged image in the region pointed out by a white rectangle in Figure 11a, shows that the surface-breaking crack had a width of 100 μm to 200 μm and a length of 20 mm to 30 mm. Figure 11b shows a picture taken from the same location of the concrete in Figure 11a after EDT. Overall, the whole concrete surface turned whiter. Furthermore, Figure 11d, an enlarged image in the region pointed out by a white rectangle in Figure 11b, clearly shows that the mouth of the surface-breaking crack was closed by white component. After SEM/EDX observation, the white components had a plate like structure and were mainly composed of magnesium and oxygen. Therefore, it can be seen that the precipitation of magnesium ions (i.e., Mg(OH)_2_) filled the mouth of the surface-breaking crack and the voids in concrete. Similar results were observed on the three other concrete samples (TP3-2, TP3-3, TP3-4) in this study. 

**Figure 12:**
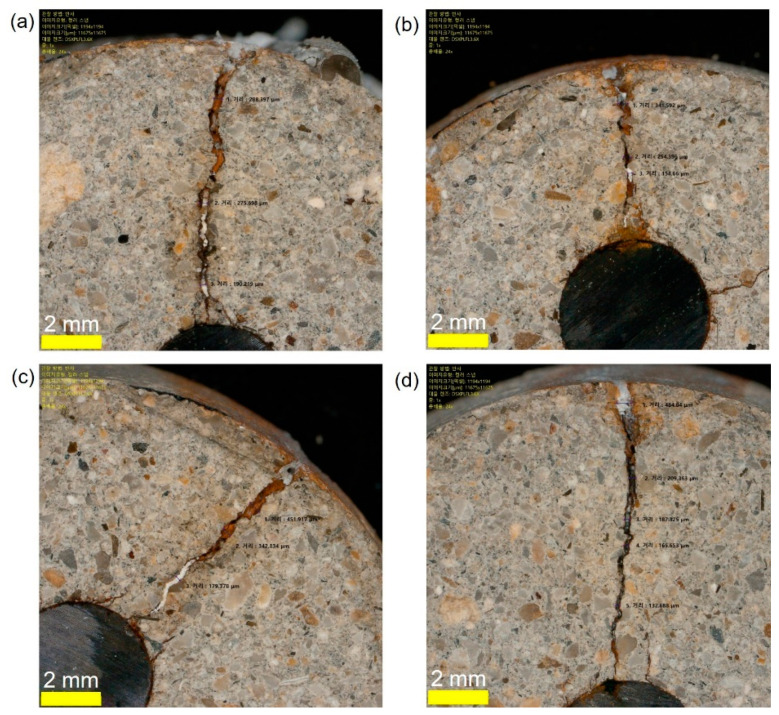
Optical microscopic images of the cross-sections of concrete samples: (**a**) TP3-1, (**b**) TP3-2, (**c**) TP3-3, and (**d**) TP3-4, respectively, after being rehabilitated by EDT with various configuration of pre and post treatment of EDT processes.

**Figure 13:**
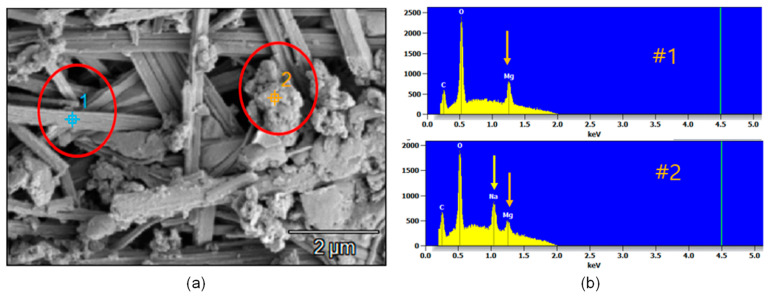
SEM/EDAX image of the cross-section of the concrete sample TP3-3 at the tip of a surface-breaking crack after EDT process: (**a**) SEM image, (**b**) distribution of primary elements in the regions 1 and 2 in the SEM image shown in Figure 13a.

**Figure 14:**
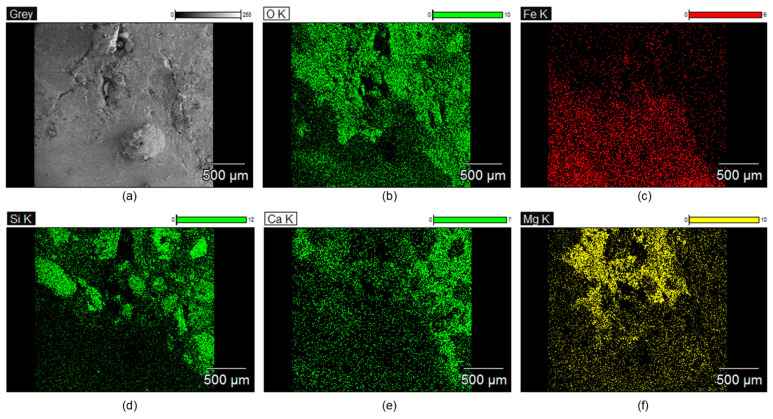
Equivalent elemental maps representing spatial distribution of: (**a**) O, (**b**) Fe, (**c**) Si, (**d**) Ca, (**e**) Ca and (**f**) Mg, respectively, measured at the tip of a surface-breaking crack in the concrete sample TP3-3 after being rehabilitated by EDT process.

Figure 12a–d show the optical images of the cross-sections of the four concrete samples after being treated by four different EDT processes, TP3-1, TP3-2, TP-3-3, and TP3-4, respectively. Precipitation of magnesium ions appeared around the mouth of surface-breaking crack and continued to the tip of the cracks along the crack interface. Cross section image shows that the precipitation of magnesium formed in the porous corrosion products, which would become a barrier to decrease the diffusion rate of dissolved oxygen and external harmful materials and thus suppress the corrosion rate. Figure 13a show the SEM image of the cross-section of a concrete sample at the tip of a surface-breaking crack (TP3-3). Equivalent elemental maps are shown in Figure 14b–f, representing distribution of O, Fe, Si, Ca, and Mg, respectively. The cross-section of a reinforcing steel is characterized by high Fe and low oxygen concentration, while the concrete cross-section is characterized by low Fe and high oxygen. The cross-section of the crack is presented as the region with high magnesium concentration. The cross-section images and the elemental maps demonstrate that precipitation of magnesium ions formed at the tip of a surface-breaking crack on the steel and concrete interface, which would improve the corrosion resistance of reinforced concrete. 

One interesting finding in this study is that the post-treatment with a NaOH solution significantly reduces the time for repairing a surface-breaking crack using EDT process. The effectiveness of the post-treatment process with high alkalinity solution can be explained by higher solubility of magnesium ions in lower pH as follows,

14 = pH + pOH^−^, K_sp_ = [Mg^2+^] [OH^−^]^2^ (8)

where K_sp_ is the solubility product of Mg(OH)_2_ (=5.61 × 10^−12^ at 25 °C) [41]. By taking a logarithm on both sides of the above equation and considering the ion product of water, Equation (8) can be modified to

log[Mg^2+^] = 28 − 2pH + logK_sp_ (9)

**Figure 15:**
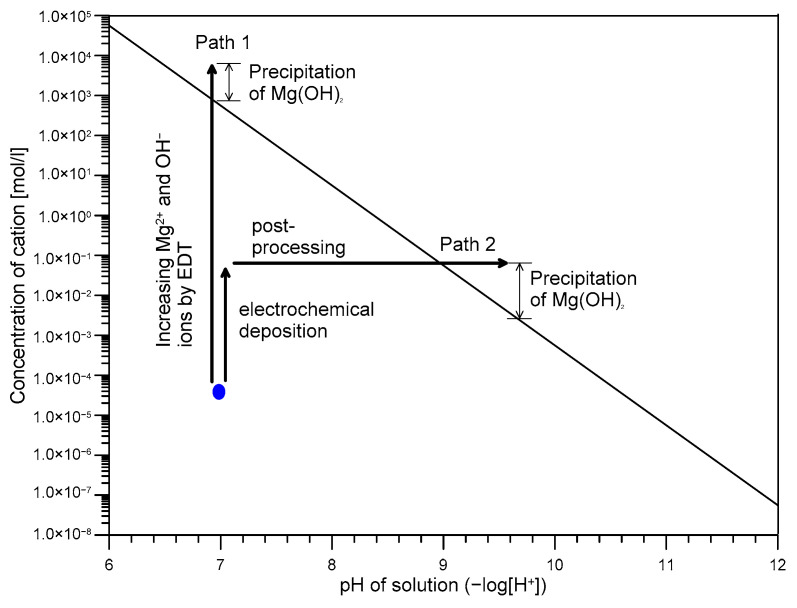
Relationship between the concentration of Mg^2+^ and the pH of the solution.

Consequently, the concentration of Mg^2+^ ions decreases as pH increases, which indicates the amount of precipitation of Mg^2+^ decreases as pH decreases. In general, the conventional EDT mechanism in the literature follows path 1 in Figure 15. The precipitation of ions is caused by reaction of magnesium ions and hydroxide ions in the vicinity of reinforcing steel in concrete, as follows,

O_2_ + 2H_2_O + 4e^−^ → 4(OH)^−^, and (10)

Mg^2+^ + 2OH^−^ → Mg(OH)_2_ + H_2_O. (11)

However, a conventional EDT process used in the experimental phases 1 and 2 was not effective for repairing (closing or filling) a surface-breaking crack in concrete samples in this study. It can be attributed to several reasons: (1) pH drop around cracks in concrete after formation of a surface-breaking crack during the accelerated corrosion test, (2) migration of (OH)^−^ ions toward the external anodic electrode during the EDT processes, and (3) pH drop in an external electrolyte due to the migration of OH^−^. It seems that only two weeks was not sufficient for conventional EDT method to work. In this study, it was found that the post treatment process with a NaOH solution is effective for facilitating the precipitation of Mg^2+^ ions through the 2nd path in Figure 15. In this light, it is not recommended to expose the repairing concrete to high alkalinity before EDT. For example, in the case of EDT process 4, the concrete specimen was treated by NaOH solution (pH 13) prior to the main electrochemical process. White precipitations were more concentrated around crack mouth, which could impede electro-deposition current flows. Consequently, less precipitations were observed around the crack tip compared to the other three concrete specimens without the pre-treatment process.

## 4. Conclusions

A series of experimental studies was conducted in this research to investigate the rehabilitation performance of electrochemical deposition treatment (EDT) for deteriorated reinforced concrete with various severity levels. Deteriorated concrete specimens, representing the three degrees of corrosion phases: corrosion initiation, rust propagation, and formation of a surface-breaking crack, were rehabilitated by a series of EDT processes. It was observed that EDT has a great potential as a comprehensive rehabilitation method for corrosion-induced deterioration with various severity levels. Specific findings and conclusions of this study are drawn as follows:

(1) It was observed from test phase 1 that EDT was an effective method to improve corrosion-resistant properties of the concrete specimens in the initiation phase of corrosion. After EDT using a 0.3 M MgCl_2_ solution in an electrolyte by applying a constant current −2 µAcm^−2^ for 24 h, impedance of the steel-and-concrete interface (i.e., charge transfer resistance and solution resistance) increased to prevent de-passivation of steel, even when the concrete specimen was exposed to harsh corrosive environment. The corrosion protection performance is attributed to precipitates on the steel surface that impeded charge transfer rate for the steel corrosion reaction (Fe → Fe^2+^ + 2e^−^) and reduction rate of dissolved oxygen (O_2_+2H_2_O +4e^−^ → 4OH^−^) in the rust layer. The use of higher concentration of MgCl_2_ solutions (i.e., 1M and 3M in this study) was effective for further decreasing the corrosion rate of steel in concrete compared to the use of 0.3M MgCl_2_ solution.
(2) Furthermore, EDT successfully suppressed the corrosion rate of concrete specimens under high corrosion activity. It was observed that the corrosion rate decreased as the polarization duration in EDT increased. The speed of corrosion rate decrement appears to be slower as the duration of EDT increases. However, it is not clear based on the limited data in this study how much the deteriorated reinforced concrete can be recovered by EDT. More systematic studies are still needed to investigate the corrosion recovery capability of EDT for concrete with various degree of corrosion levels.
(3) From the experimental results of test phase 3, EDT was effective for repairing (closing and filling) a corrosion-induced surface-breaking crack in concrete. From the visual inspection based on optical microscopic and SEM images, precipitation of magnesium ions formed at the mouth of a surface-breaking crack and continued to the crack interface and rust products, eventually reaching the crack tip rooted on the steel-and-concrete interface. One interesting finding in this study is that the post-treatment with a NaOH solution significantly reduced the time for repairing a surface-breaking crack using an EDT process.
(4) Experiments in this study were performed using reduced concrete samples and deteriorated by accelerated corrosion tests in the laboratory. More systematic studies are still needed to better understand the rehabilitation performance EDT for the deteriorated concrete in actual reinforced concrete structures.

## Figures and Tables

**Table 1:** Summary of accelerated corrosion test methods to prepare damaged concrete samples with three different target corrosion levels (corrosion initiation, rust propagation, and damage acceleration phases) for the experimental phases 1, 2 and 3, respectively.

Test Phase	Specimen No.	Method	Impressed Current		Simulated Corrosion Phase (Target Damage)
			Density	Duration	
TP1	TP1-1	Exposing specimens to 45 cycles of wetting-and-drying environment	X	X	Initiation phase (de-passivation of steel and low corrosion activity)
	TP1-2				
	TP1-3				
	TP1-4				
TP2	TP2-1	Using the impressed current technique	X	X	Solid
	TP2-2		0.5 μA/cm^2^	24 h	Rust propagation phase (high corrosion activity)
	TP2-3				
	TP2-4				
TP3	TP3-1		1 mA/cm^2^	24 h	Damage acceleration phase (formation of a surface-breaking crack)
	TP3-2				
	TP3-3				
	TP3-4				

**Table 2:** Summary of electrochemical deposition treatment (EDT) processes used in test phases 1, 2 and 3.

Test Phase	Specimen No.	Pre-Treatment	Deposition			Post-Treatment	Deposition	Post-Treatment	Baking
			Electrolyte	Current	Duration				
		①	②			③	④	⑤	⑥
TP1	TP1-1	X	X	X	X	X	X	X	60 °C 5 h
	TP1-2		0.3 M MgCl_2_	−2 μA/cm^2^	24 h	X	X	X	
	TP1-3		1.0 M MgCl_2_						
	TP1-4		3.0 M MgCl_2_						
TP2	TP2-1	X	X	X	X	X	X	X	
	TP2-2		X	X	X				
	TP2-3		2.0 M MgCl_2_	−2 μA/cm^2^	48 h				
	TP2-4				168 h				
TP3	TP3-1	X	2.0 M MgCl_2_	−2 μA/cm^2^	240 h (10 days)	NaOH (pH 13)	X	X	
	TP3-2						O	X	
	TP3-3						O	O	
	TP3-4	NaOH (pH 13)					X	X	

**Table 3:** Fitting parameters used for curve-fitting in Figure 9a,b.

Specimen No.	Fitting Parameters									Two-Frequency Values		
	*R*_0_ kΩcm^2^	*C*_1_ μFcm^−2^	*α*_1_	*R_f_* kΩcm^2^	*C*_2_ μFcm^−2^	*α*_2_	*R_c_* kΩcm^2^	*W*	*R_p_* kΩcm^2^	*Z_low_* kΩcm^2^	*Z_high_* kΩcm^2^	$R_{P}^{\prime }$ $k\Omega {\mathrm{cm}}^{2}$
TP2-1	5.23 × 10^−2^	1.13 × 10^−7^	0.91	2.62 × 10^−2^	3.10 × 10^−4^	0.85	2.06 × 10^9^	-	8.37 × 10^14^	244.09	0.07	244.02
TP2-2	7.85 × 10^−2^	5.40 × 10^−7^	0.66	7.28 × 10^−2^	5.20 × 10^−4^	0.78	19.5	1.59 × 10^0^	14.7	15.24	0.06	15.18
TP2-3	1.45 × 10^−2^	3.76 × 10^−7^	0.73	7.02 × 10^−2^	1.10 × 10^−3^	0.71	38.5	1.34 × 10^−6^	37.56	29.82	0.10	29.72
TP2-4	0.80 × 10^−2^	7.07 × 10^−9^	0.83	1.82 × 10^0^	5.10 × 10^−4^	0.77	165.10	2.60 × 10^−4^	135.72	92.23	1.59	90.64

## Data Availability

Data are contained within the article. However, the data presented in this study are also available on request from the corresponding author.
